# Insight into the role of alternative splicing within the RBM10v1 exon 10 tandem donor site

**DOI:** 10.1186/s13104-015-0983-5

**Published:** 2015-02-19

**Authors:** Sarah J Tessier, Julie J Loiselle, Anne McBain, Celine Pullen, Benjamin W Koenderink, Justin G Roy, Leslie C Sutherland

**Affiliations:** Department of Biology, Laurentian University, 935 Ramsey Lake Road, Sudbury, ON P3E 2C6 Canada; Biomolecular Sciences Program, Laurentian University, 935 Ramsey Lake Road, Sudbury, ON P3E 2C6 Canada; Genetics Lab, Health Sciences North, 41 Ramsey Lake Road, Sudbury, ON P3E 5 J1 Canada; AMRIC, Health Sciences North, 41 Ramsey Lake Road, Sudbury, ON P3E 5 J1 Canada; Department of Chemistry and Biochemistry, Laurentian University, 935 Ramsey Lake Road, Sudbury, ON P3E 2C6 Canada; Division of Medical Sciences, Northern Ontario School of Medicine, Laurentian University, 935 Ramsey Lake Road, Sudbury, ON P3E 2C6 Canada; Department of Medicine, Division of Medical Oncology, University of Ottawa, Ottawa, ON Canada

**Keywords:** RBM10, RNA binding protein, RRM, Alternative splicing, Regulation, NUMB

## Abstract

**Background:**

RBM10 is an RNA binding protein involved in the regulation of transcription, alternative splicing and message stabilization. Mutations in *RBM10*, which maps to the X chromosome, are associated with TARP syndrome, lung and pancreatic cancers. Two predominant isoforms of RBM10 exist, RBM10v1 and RBM10v2. Both variants have alternate isoforms that differ by one valine residue, at amino acid 354 (RBM10v1) or 277 (RBM10v2). It was recently observed that a novel point mutation at amino acid 354 of RBM10v1, replacing valine with glutamic acid, correlated with preferential expression of an exon 11 inclusion variant of the proliferation regulatory protein NUMB, which is upregulated in lung cancer.

**Findings:**

We demonstrate, using the GLC20 male-derived small cell lung cancer cell line - confirmed to have only one X chromosome - that the two (+/−) valine isoforms of RBM10v1 and RBM10v2 result from alternative splicing. Protein modeling of the RNA Recognition Motif (RRM) within which the alteration occurs, shows that the presence of valine inhibits the formation of one of the two α-helices associated with RRM tertiary structure, whereas the absence of valine supports the α-helical configuration. We then show 2-fold elevated expression of the transcripts encoding the minus valine RBM10v1 isoform in GLC20 cells, compared to those encoding the plus valine isoform. This expression correlates with preferential expression of the lung cancer-associated NUMB exon 11 inclusion variant.

**Conclusions:**

Our observations suggest that the ability of RBM10v1 to regulate alternative splicing depends, at least in part, on a structural alteration within the second RRM domain, which influences whether RBM10v1 functions to support or repress splicing. A model is presented.

## Findings

### Background

The RNA binding protein RBM10 is capable of regulating the expression of a number of genes including some involved in apoptosis (e.g., FASR) [1] and cell proliferation (e.g., NUMB) [2]. In all of the reported cases but one, this expression regulation takes the form of alternative splicing regulation [1-4]. The one gene whose expression is not regulated by RBM10-mediated alternative splicing is the angiotensin receptor 1 (AT1), where RBM10 influences AT1 expression by binding within the transcript’s 3′-untranslated region (UTR), stabilising the message and subsequently contributing to a decreased rate of transcription [4]. How RBM10 accomplishes these disparate functions remains to be determined.

The region(s) within the RBM10 protein that is involved in any of the RNA-protein interactions identified to date has not been defined although it likely involves at least one of two RNA Recognition Motifs (RRM) and two zinc fingers (ZnF) [5]. On the other hand, two consensus motifs within the target RNA with which the RBM10 protein binds have been defined as CUCUGAACUC and CGAUCCCU [2]. Using HeLa cells, Bechara et al. [2] reported that RBM10 interactions occurred predominantly within the upstream intron of an excluded alternate exon but predominantly downstream of an included alternate exon. On the other hand, using HEK293 cells, Wang et al. [3] reported that RBM10 interactions occurred in the vicinity of the 5′- and 3′-splice sites within both the upstream and downstream introns of alternate exons, though predominant binding in the vicinity of the upstream 3′-splice site was associated with less exon skipping. Obviously, much remains to be elucidated concerning the regulation of these interactions.

Mutations in RBM10 have been noted in cells associated with lung and pancreatic cancers and the neuromuscular disorder TARP syndrome [3,6-10] (summarized in Table 1). In one lung study, 12/183 (7%) adenocarcinoma specimens had RBM10 mutations, each patient having a different mutation (5/12 being missense, 5/12 truncating and 2/12 splice-site) [10]. In another lung study, a mutation - caused by a T to A substitution and consequent valine (V) to glutamic acid (E) substitution within the second RRM (RRM2) of RBM10 - was reported in A549 lung adenocarcinoma cells, with consequences for NUMB alternative splicing [2]. In the blood cells of patients with TARP syndrome, six different mutations were identified [6-8], and a six exon-spanning deletion (aa 651–889) that codes for a truncated RBM10 isoform with an inability to regulate alternative splicing [3].

**Table 1:** **RBM10 mutations**

**Phenotype**	**Mutation effect**	**Mutation**	**Exon**	**Protein**	**Reference**
NSCLC	Missense		2	E4K	Imielinski et al. [10]
NSCLC	Missense		2	R6H	Imielinski et al. [10]
TARP syndrome	Frameshift	c.159delC	3	p.Lys54SerfsX80	Gripp et al. [7]
NSCLC	Nonsense		3	E67	Imielinski et al. [10]
TARP syndrome		c.448C>T	4	p.Gln150X	Johnston et al. [8]
NSCLC	Nonsense		5	R157fs	Imielinski et al. [10]
NSCLC	Nonsense		7	Y206	Imielinski et al. [10]
NSCLC	Nonsense		8	R230	Imielinski et al. [10]
TARP syndrome		c.724+2T>C	8		Johnston et al. [8]
NSCLC	Missense		10	1316F	Imielinski et al. [10]
TARP syndrome	Nonsense	c.1235G>A	12	p.Trp412X	Johnston et al. [6]
NSCLC	Missense		16	Y580F	Imielinski et al. [10]
NSCLC	Splice site		17	Y596	Imielinski et al. [10]
Pancreatic neoplasm	Frameshift	c.1817-1818insA	17	p.E606EfsX37	Furukawa et al. [9]
TARP syndrome	Frameshift	c.1893-1894insA	17	p.Pro632ThrfsX41	Johnston et al. [6]
TARP syndrome	Deletion	aa651-889	18-23		Wang et al. [3]
NSCLC	Missense		18	R685L	Imielnski et al. [10]
TARP syndrome		c.2176C>T	20	p.Arg726X	Johnston et al. [8]
NSCLC	Nonsense		21	E810	Imielinski et al. [10]
NSCLC	Splice site		22	V846	Imielinski et al. [10]

The pre-mRNA for RBM10 is alternatively spliced to yield two predominant protein isoforms: a 930 amino acid (aa) (~103.5 kDa) isoform that includes exon 4, referred to as RBM10 variant 1 (RBM10v1), and an 853 aa (~94.5 kDa) isoform that lacks exon 4, referred to as RBM10v2. In addition to the two major RBM10 variants, the Ensembl Database lists a variant of RBM10v2 that is one amino acid shorter (852 aa). The 7 April 2003 GenBank deposition version 1 for this RBM10v2(V277del) isoform (NM_152856.1) describes it as having an “alternate donor splice site” compared to RBM10v1. Indeed, a tandem donor splice site of the configuration GYNGYN was identified in RBM10 exon 10 (as GTGGTG) from a screen of expressed sequence tags (ESTs) for variants generated from upstream and downstream tandem repeat triplets [11]. Utilization of the downstream triplate (the “e” site referred to in [11]) would result in the inclusion of a valine at amino acid 277 for RBM10v2 and 354 for RBM10v1. For clarity throughout this manuscript, we will refer to the longer RBM10v2 isoform as RBM10v2(V277), the shorter RBM10v2 isoform as RBM10v2(V277del), the longer RBM10v1 isoform as RBM10v1(V354) and the shorter RBM10v1 isoform as RBM10v1(V354del).

Our comprehensive review of the literature and various protein databases suggested that the presence of RBM10v1(V354del) is by no means generally recognized and that there appears to be some confusion as to its legitimacy and the mechanism by which it is generated. For instance, RBM10v1(V354del) has been identified as RBM10 “missing” valine 354 (UniProt – P98175-2), and as a mismatch of I353 with valine (on the Protein Data Bank website). For an overview of RBM10v1 isoforms described in various databases and references, see Table 2.

**Table 2:** **RBM10v1 isoforms reported in various database and references**

**Origin**	**Sequence**	**Reference**
**Databases**		
Ensembl	STIVEAA	ENST00000377604
NIH GenBank	STIVEAA	NM_005676
	STIEAA	NM_001204467
NIH GenBank	STIVEAA	NP_005667
	STIEAA	NP_001191396
EMBL-EBI InterPro	STIVEAA	P98175
UniProtKB	STIVEAA	P98175-1
	STI-EAA	P98175-2
neXtprot beta	STIVEAA	iso1
	STI-EAA	iso2
USCSC	STIVEAA	uc004dhf.3
	STI-EAA	uc004dhh.3
HUGE	STI-EAA	KIAA0122/GenBank D50912
**References**		
Bechara et al. [2]	Minus valine isoform; STI(E)EAA	
Inoue et al. [1]	STI-EAA	KIAA0122

In the study presented herein using a small cell lung cancer cell line, we confirm the existence of two full-length RBM10v2 transcripts, RBM10v2(V277) and RBM10v2(V277del), and prove the existence of two full-length RBM10v1 transcripts, RBM10v1(V354) and RBM10v1(V354del). We then demonstrate that the presence or absence of valine alters the tertiary structure of the second RNA Recognition Motif (RRM2) within the RBM10 protein. Finally, we show that 2-fold higher levels of the transcripts encoding the minus - compared to the plus - valine isoforms of both RBM10v1 and RBM10v2 correlates with higher levels of the lung cancer associated NUMB exon 11 inclusion variant, compared to the exon 11 exclusion variant.

## Results

### Evidence that alternative splicing occurs within exon 10

**Figure 1:**
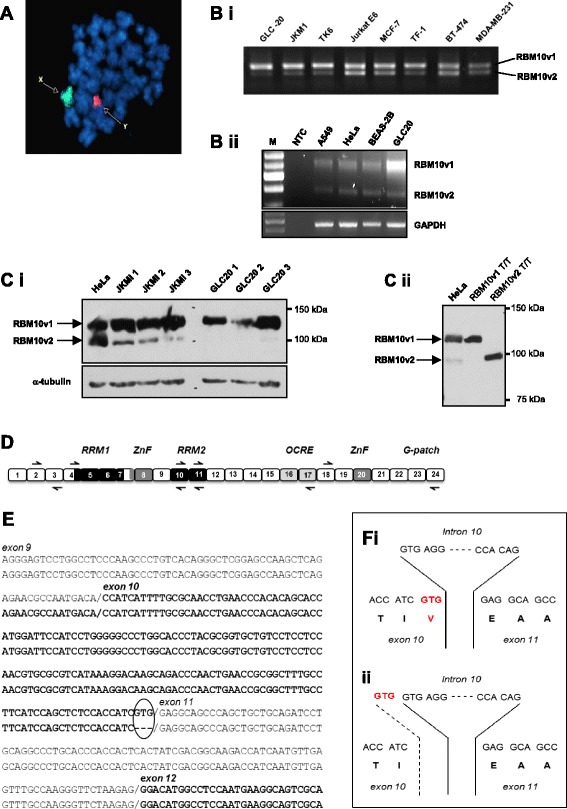
**Alternative splicing of RBM10. (A)** FISH analysis of GLC20 cells, with painted X and Y chromosomes, demonstrating the presence of only one X chromosome. **(B)** RBM10v1 and RBM10v2 RNA expression in various cell lines, including GLC20. Representative raw RT-PCR data using RBM10 exon 4 spanning primers. *Bi*: RBM10F with RBM10RS primers. *Bii*: RBM10F with RBM10v1/v2R primers. M: 100 bp DNA ladder (FroggaBio Inc., Toronto, Canada). NTC: no template control. **(C)** Protein expression by Western blot. *Ci* shows RBM10 expression in whole cell lysates from three cell lines, including GLC20. The numbers 1, 2 and 3 after JKM1 and GLC20 delineate cells from three biological replicates. *Cii* includes control HeLa protein and *in vitro* translated RBM10v1 and RBM10v2 protein, to confirm the location of RBM10v2 is the cell line extracts. **(D)** Cartoon of full-length RBM10v1 mRNA, not drawn to scale. Boxes represent exons. Left and right black arrows represent primer placement for sequencing. Approximate positioning of consensus functional motifs is indicated by text and differential shading. **(E)** Alignment of the two GLC20 RBM10v1 isoform sequences. Circled area indicates the region that differs between the two RBM10v1 isoforms. **(F)** Nucleotide and amino acid sequences of the RBM10v1 exon10/intron 10/exon 11 donor and acceptor sites for *(i)* RBM10v1(V354), and *(ii)* RBM10v1(V354del).

The GLC20 cell line was established from a small cell lung cancer (SCLC) of male origin [12]. It is RBM5-null and was determined, by FISH, to contain only one X chromosome (Figure 1A). Alternative splicing of the X-linked RBM10 gene [13], to generate both RBM10v1 and RBM10v2, was confirmed in the GLC20 cells, using RT-PCR (Figure 1B). Most of the cancer, or transformed, cell lines that we have tested express more RBM10v1 than RBM10v2, at both the mRNA (Figure 1B) and protein (Figure 1C and data not shown) levels. This observation is particularly true for GLC20 cells, which express such a small amount of RBM10v2 protein that it is technically challenging to detect.

To estimate functionality of RBM10 in the GLC20 cells, sequencing of cDNA was carried out. Only full-length RBM10v1 cDNA was sequenced because full-length RBM10v2 cDNA could not be amplified from the GLC20 cells. Mixed sequence was observed beginning at the 3′-end of exon 10, with either the forward or reverse primer. At that point in time (June 2014), the Ensembl Database listed two different RBM10v2 isoforms, we are herein designating RBM10v2(V277) and RBM10v2(V277del) (corresponding to GenBank Accession Numbers NM_001204466.1 and NM_152856.2). To determine if the mixed sequencing read in the GLC20 RBM10v1 sample was the result of a mixture of transcripts with different exon 10 3′-end sequences, we designed primers immediately 3′- to the anticipated modified triplet site in the cDNA (refer to Figure 1D for primer locations), thereafter expecting an unambiguous sequence read. Indeed, clear sequence was obtained, thereby delineating the altered region as the last three nucleotides of exon 10 (Figure 1E).

The presence of a mixture of two different isoforms of RBM10v1 suggested either an alternative splicing event or two *RBM10* alleles, the later possibility being unlikely since we had confirmed only one X chromosome by FISH analysis (Figure 1A). Since there remained the possibility of an RBM10 gene duplication with allelic variation, we decided to sequence RBM10v1 cDNA from additional cell lines, theorizing that if the same mixed read was observed in other cells it would suggest alternative splicing. We sequenced transcripts from both male and female-derived cell lines, since one *RBM10* allele is silenced as the result of X chromosome inactivation in female somatic cells [14,15], therefore whether male or female-derived, cell lines would theoretically have only one *RBM10* gene that is transcribed. RBM10v1 and RBM10v2 cDNA was sequenced in A549 (a male-derived lung adenocarcinoma cell line), HeLa (a female-derived cervical adenocarcinoma cell line) and BEAS-2B (a male-derived non-cancerous SV40/adenovirus-transformed lung cell line). Sequencing data revealed the same nucleotide variation in exon 10 of RBM10v1 and exon 9 of RBM10v2 in all three cell lines, therefore suggesting that the two isoforms of RBM10v1 are, as originally surmised within GenBank deposition NM_152856.1 and by Hiller et al. [11], the result of an alternative splicing event. An example of how this might occur is presented diagrammatically in Figure 1F.

Sequence variation that occurs as a result of alternative splicing at the 3′-ends of exons with tandem repeats such as GYNGYN, is regulated partly by U1snRNA binding and partly by the presence of CGGG and GGGT sequons in the downstream intron, particularly if the downstream intron is shorter than 200 nucleotides (nts) [11]. Intron 10 of RBM10 is 171 nts and contains three CGGG sequons, two GGGT sequons and one CGGGGT sequon, suggesting that co-expression of the two RBM10v1 isoforms is indeed a regulated alternative splicing event. The stimuli that control this regulation remain to be determined, but are unlikely limited to male, lung or cancer cells, since both RBM10v1(V354) and RBM10v1(V354del) were present in A549, HeLa, BEAS-2B and GLC20 cells.

### Structural consequences of alternative splicing of RBM10v1

RBM10v1 and RBM10v2 both contain two RRMs (refer to Figure 1D) defined as ~75-85 amino acids that three dimensionally form four β-sheets flanked by two α-helices. The most conserved sequences within any RRM comprise the RNP2 and RNP1 domains (reviewed in [5]), RNP1 having the most highly conserved sequence of the two. Notably, amino acid 354 in RBM10v1 is located in approximately the middle of RRM2, at the beginning of the second α-helix and near to the end of β-sheet three, which is encoded by the highly conserved RNP1 domain [16].

Valines have a high free energy that is associated with α-helix disruption and since α-helices are known to contribute to the stabilization of RNA/protein interactions, disruption of the α-helix by a valine insertion would be predicted to destabilize any RNA/protein interaction. In RBM10, the placement of the altered amino acid adjacent to the highly conserved β-sheet three structure suggested that any potential alteration to the second α-helical structure within RRM2 might have a significant effect on overall protein conformation, and consequently, the ability to interact with RNA. Indeed, Bechara et al. [2] recently described a G to T mutation in the terminal exon 10 codon of RBM10, which changed the amino acid coded from valine (V) to a glutamic acid (E). This single mutation had dramatic functional consequences, manifesting as an altered ability to splice specific downstream targets, such as pre-mRNA encoding the proliferation regulatory protein NUMB [2].

**Figure 2:**
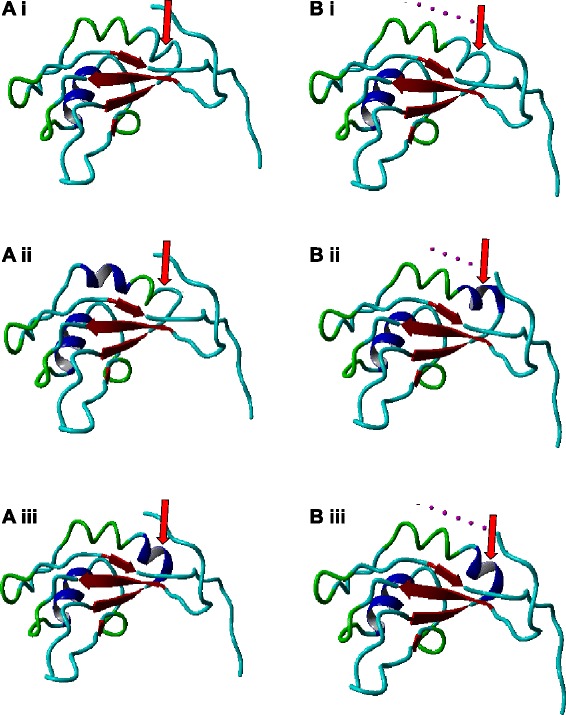
**Conformation of RBM10v1 RRM2.** The RBM10 RRM2 conformation was modeled using SwissProtKB **(A)** or Phyre^2^ **(B)** *. (i)* Isoforms V354. *(ii)* Isoforms V354E. *(iii)* Isoforms V354del*.* Arrow indicates the position of the +/− valine. Yasara structure colors indicate beta-sheets (red), alpha-helixes (dark blue), turns (green) and random coils (cyan).

To better understand how a change to this residue might contribute to RBM10 functional alterations we compared the structures of a valine-retaining, a glutamic acid-substituting and a valine-lacking amino acid within RRM2. We uploaded these altered RBM10v1 RRM2 sequences into SwissProtKB/Swiss-Prot (www.expasy.org), a program that predicts a two-dimensional configuration and ranks it against similar configurations of previously crystalized structures. A crystal structure for a minus-valine RBM10 RRM2 (designated 2m2d) [17,18] was the reference structure for the V354, V354E and V354del RBM10v1 RRM2 predictions. We also uploaded the RRM2 sequences into Phyre^2^ (the Protein Homology/analogY Recognition Engine v2.0, www.sbg.bio.ic.ac.uk). To visualize a rotatable three-dimensional structure, the structure predictions for V354, V354E and V354del from both SwissProtKB and Phyre^2^ were uploaded into the Yasara modeling program (Yet Another Scientific Artificial Reality Application, www.yasara.org). A comparison of all the predictions (Figure 2) revealed that the addition of valine did, as anticipated, disrupt the α-helical structure and thus the classic configuration of an RRM domain (shown as a colour change from dark blue to cyan by the Yasara software). Exclusion of the valine was associated with an α-helix. Substitution of the V for an E resulted in two slightly different configurations, depending on the prediction program used: both programs, however, predicted a change to, but a retention of, an α-helical structure compared to either the V354del or the V354. These modelling results suggest that conformational changes to the RBM10v1 protein could be responsible for altering the protein’s ability to interact with RNA.

### Functional consequences of alternative splicing of RBM10v1

Using PAR-CLIP technology Bechara et al. [2] identified an RBM10 cluster in the 3′-splice site region preceding NUMB exon 11 (the current Ensembl exonic designation for the previously referred to NUMB exon 9) [2]. They then went on to demonstrate, using an A549 non-small cell lung cancer (NSCLC) cell line with an RBM10v1(V354E) mutation (herein referred to as A549-JV), that expression of recombinant RBM10v1 protein, with either a valine or a glutamic acid at amino acid 354, altered NUMB splicing. With valine present (RBM10v1(V354)), there was preferential NUMB exon 11 exclusion - associated with NOTCH repression and decreased proliferation. On the other hand, the glutamic acid-containing isoform (RBM10v1(V354E)) demonstrated preferential NUMB exon 11 inclusion – associated with NOTCH activation and increased proliferation. Recognising that a V354E substitution would not necessarily have a similar effect as a V354del, but taking into consideration our structural predictions, the fact that the valine to glutamic acid mutation occurred at exactly the same site as the RBM10v1 alternative splicing of RBM10v1(V354) to RBM10v1(V354del) suggested to us that regulated alternative splicing of RBM10v1 has functional significance and is important to lung cancer.

**Figure 3:**
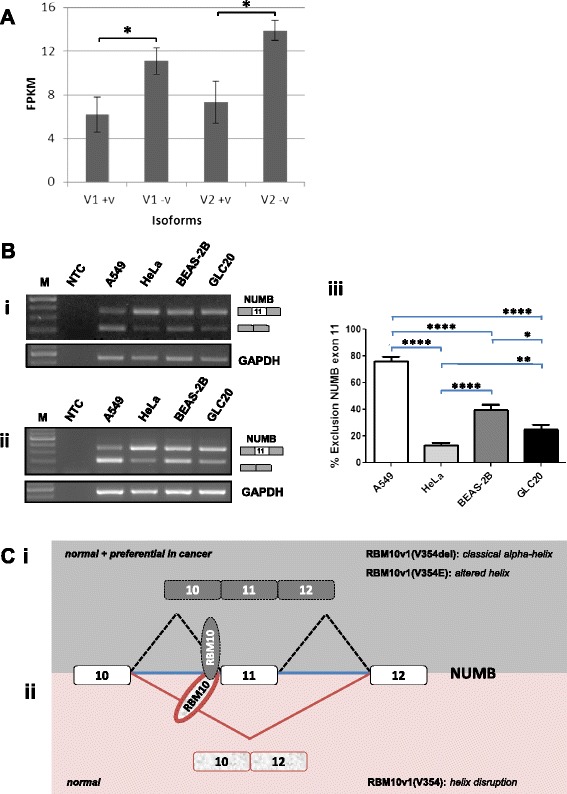
**Functional affects associated with RBM10 variant expression. (A)** In GLC20 cells, comparative expression levels of RBM10v1 and RBM10v2 transcripts encoding the valine-retaining and valine-lacking isoforms, as determined by RNA-seq. *p < 0.05. **(B)** NUMB alternative splicing in A549, HeLa, BEAS-2B and GLC20 cells. *(i)* and *(ii)* are 2% agarose gels with SYBR®safe, showing representative amplicon expression levels following end-point PCR with *(i)* 28 cycles, or *(ii)* 40 cycles, using NUMB exon 11-spanning primers and *(i)* 19 cycles or *(ii)* 25 cycles for GAPDH. *(iii)* Following densitometry of amplicons from six end-point PCR reactions (three at 28 cycles and three at 40 cycles) of the two different cDNA preparations from one RNA extraction for each cell line, the percentage of the NUMB exon 11 exclusion variant was calculated and plotted. Error bars represent the standard error of the mean. Significances were calculated using an unpaired Student’s *t*-test, with *p < 0.05, **p < 0.01 and ****p < 0.0001. **(C)** Model depicting how the +/− valine isoforms of RBM10v1 might influence NUMB exon 11 alternative splicing. The plus and minus valine isoforms are present, at low levels, in normal cells, and contribute to the production of both the exon 11 inclusion and exclusion variants of NUMB. Both RBM10 isoforms are able to bind NUMB pre-mRNA, but the minus valine isoform of RBM10v1 does so with higher affinity, having the classical α-helix structure. The plus valine isoform does not bind as efficiently, thereby interfering with recognition of the intron 10 3′splice site, resulting in NUMB exon 11 exclusion.

Considering that RBM10v1(V354) expression correlated with preferential NUMB exon 11 exclusion and RBM10v1(V354E) expression correlated with preferential NUMB exon 11 inclusion [2] and that expression of the exon 11 retaining NUMB transcript is frequently increased in lung adenocarcinomas [19], one might predict that (a) downregulation of RBM10v1(V354) is one means by which lung cancer cells circumvent proliferation controls, and (b) more RBM10v1(V354del) than RBM10v1(V354) is expressed in lung cancers. Interestingly, we have whole transcriptome-sequencing data (manuscript in preparation) demonstrating that in the GLC20 small cell lung cancer cells and three stable GLC20 sublines, transcripts encoding RBM10v1(V354del) and RBM10v2(V277del) have ~2-fold higher expression than transcripts encoding RBM10v1(V354) and RBM10v2(V277) (Figure 3A). When NUMB exon 11 alternative splicing was examined in the GLC20 cells, as predicted, preferential expression of the NUMB exon 11 inclusion variant was observed (Figure 3B).

NUMB splicing was also examined in HeLa and BEAS-2B cells as well as our A549 cells (herein referred to as A549-LS) that, unlike those used by Valcárcel and colleagues [2], express transcripts encoding both RBM10v1(V354) and RBM10v1(V354del), as opposed to only RBM10v1(V354E). If RBM10v1(V354del) functions in a similar manner to RBM10v1(V354E) - to generate the NUMB exon 11 inclusion transcript - then we anticipated A549-LS cells would express both the NUMB exon 11 inclusion and exclusion transcripts. We also anticipated more of the NUMB exon 11 exclusion transcript in the A549-LS cells (resulting from RBM10v1(V354) expression), compared to the A549-JV subline (which lacks RBM10v1(V354)). As shown in Figure 3B, and confirming the observations of Valcárcel and colleagues [2], the A549-LS subline expressed both NUMB variants, but significantly more of the NUMB exclusion variant than the inclusion variant (as opposed to the A549-JV subline that expressed predominantly the NUMB exon 11-inclusion transcript: see [2], Figure Six C, upper panel, lane 14). In the transformed but non-tumourigenic BEAS-2B bronchial epithelial cells, we predicted a lower NUMB exon 11 inclusion to exclusion ratio, based on the previously reported observations that the ratio of NUMB exon 11 inclusion to exon 11 exclusion is higher in lung cancer than in normal cells [19] and our hypothesis that the RBM10v1(V354del) isoform is more prevalent in cancer cells. As shown in Figure 3B, the BEAS-2B cells did indeed have the lowest NUMB exon 11 inclusion to exclusion ratio of the four cell lines examined. Finally, as expected, and previously shown by Bechara et al. [2], HeLa cells had a clearly higher inclusion to exclusion expression ratio. The only unexpected observation from this cell line RNA analysis was the high level of the NUMB exon 11 exclusion variant in the A549-LS cells, considering they are a lung cancer cell line. Determination of the relative expression levels of the transcripts encoding RBM10v1(V354) and RBM10v1(V354del) isoforms in this subline would help to resolve this conundrum.

## Discussion

RBM10 is an apoptosis regulatory protein [20] and its paralogue, RBM5 functions in the same capacity, but has the added ability to regulate the cell cycle [21,22]. Based on the findings of Bechara et al. [2], it appears that RBM10 is also able to regulate the cell cycle, via the NOTCH signaling pathway [2]. Is the regulation of RBM10v1(V354) versus RBM10v1(V354del) alternative splicing a means by which normal cells temporarily modulate proliferation prior to a repair versus apoptosis event? Have cancer cells hijacked this alternative splicing mechanism as one means of circumventing proliferation controls? Of the mutations identified so far in pancreatic cancer and NSCLCs, none occurs at this site to permanently generate the minus valine isoform, except the mutation described by Bechara et al. [2] in a lung adenocarcinoma cell line (see Table 1). It is therefore difficult to predict how important the regulation of this alternative splicing event is to cancer initiation and/or progression. We have initiated studies to more thoroughly characterise the expression of transcripts encoding the two RBM10v1 and RBM10v2 isoforms in primary lung cancer specimens. Additional studies relating to protein expression are also warranted, as are studies concerning the regulation of this expression.

In an attempt to align our observations with those of Bechara et al. [2] and Wang et al. [3] we propose a model to explain how changes in RBM10v1 splicing might be predicted to regulate NUMB splicing. The model takes into consideration the following observations. Firstly, overexpression of RBM10 correlates with NUMB exon 11 exclusion [2,3]. Secondly, in lung cancer there is preferential expression of the NUMB exon 11 inclusion variant. Thirdly, in lung cancer RBM10 is highly expressed [10], but while in our A549, HeLa, BEAS-2B and GLC20 cells transcripts encoding both RBM10v1 isoforms were observed, there was two-fold more RBM10v1(V354del) than RBM10v1(V354) in the GLC20 SCLC cells. Our model does not attempt to reconcile the somewhat conflicting binding data previously reported [2,3].

Our model, depicted in Figure 3C, posits that RBM10v1(V354) and RBM10v1(V354del) are both expressed in normal cells, but that in cancers RBM10v1(V354del) is preferentially expressed. RBM10v1(V354) and RBM10v1(V354del) are both capable of interacting with pre-mRNA, and do so in the vicinity of the upstream intronic 3′-splice site of the alternate exon. RBM10v1(V354del), however, has a higher affinity interaction with pre-mRNA than RBM10v1(V354) (resulting from its ability to form the second α-helix within RRM2). In its capacity as a higher affinity binder, RBM10v1(V354del) is able to function as an auxiliary splicing factor. As for RBM10v1(V354), disruption of the second α-helical structure within RRM2 generates an RBM10v1 isoform that interacts with the same pre-mRNA as RBM10v1(V354del) but in a manner that impedes splicing. As a result of either less efficient or lower affinity binding or an inability to optimally interact with other auxiliary/splicing factors, expression of RBM10v1(V354) functions to increase the splicing of the alternative transcript. In our model, the increase in the NUMB exon 11 inclusion transcript that is associated with downregulation of RBM10 results from the presence of reduced levels of the competing, less efficient binding, RBM10v1(V354) isoform and consequently more binding of the higher affinity RBM10v1(V354del) isoform, resulting in more of the NUMB exon 11 inclusion product. According to our model, therefore, alternative splicing regulation by RBM10 depends not only on the ratio of RBM10v1(V354) to RBM10v1(V354del), but on the total levels of RBM10v1 protein as well.

Exactly how the structural change associated with a valine insertion at amino acid 354 of RBM10v1 results in splicing changes is unknown. Multiple binding motifs have been identified for RBM10 [2] as have multiple potential binding regions within pre-mRNA [2,3], suggesting that interaction of RBM10 with pre-mRNA targets may be a dynamic and flexible phenomenon. And while expression of RBM10 is more frequently associated with alternate exon exclusion [1-3,23], it is also associated with alternate exon inclusion [2,3]. Perhaps binding and function of RBM10 is more influenced by tertiary structure than primary sequence of the protein, and the multiple sequence motifs and binding regions identified within pre-mRNAs reflect interactions with different RBM10 isoforms that each assume a slightly different conformation.

To note just prior to submission of this manuscript, Ensembl (which had not, at least during the course of this investigation, listed RBM10v1(V354del) in its database) removed the RBM10v2(V277del) isoform, thereby eliminating all reference to the minus-valine isoforms. The minus valine isoforms are those which form the classic α-helical structure associated with the RRM domains (as shown herein), RNA transcripts encoding the minus valine isoform of RBM10v1 exist in multiple cell types (as shown herein), and functional studies demonstrate the apoptotic regulatory ability [20] and alternative splicing ability [2] of RBM10v1(V354del). We therefore suggest that the alternative splicing of RBM10v1, and likely RBM10v2, is a regulated event worthy of consideration in functional studies.

## Materials and methods

### Cell culture and differentiation

GLC20 cells were kindly provided by Charles Buys (University of Groningen, The Netherlands). Cells were cultured in RPMI supplemented with 10% fetal bovine serum (FBS). JKM1 [24], Jurkat Clone E6 [25], MCF-7 [26], MDA-MB-231 [27] and TF-1 cells [25] were grown as previously described. A549 (from ATCC) and HeLa cells (provided by Hoyun Lee, AMRIC) were grown in DMEM/F-12 medium supplemented with 10% FBS. TK6 cells (provided by Elliot Drobetsky, University of Montreal) were grown in DMEM/F-12 medium supplemented with 10% fetal horse serum. BEAS-2B cells (purchased from ATCC) were grown in LHC-9 medium, on plates pre-coated with 0.01 mg/ml fibronectin, 0.03 mg/ml collagen and 0.01 mg/ml bovine serum albumin. Media and sera were purchased from Life Technologies (Burlington, Canada).

### Fluorescent in situ hybridization (FISH)

Slides were prepared from a cell suspension using standard cytogenetic techniques. Slides were denatured at 75°C for 2 mins and hybridized overnight at 37°C with Spectrum Green (X chromosome) and Spectrum Red (Y chromosome) (Vysis, Abbott Molecular, Mississauga, Canada). Following hybridization the slides were washed in 0.4×SSC/0.3% NP40 at 73°C for 2 mins, then 2.0×SSC/0.1% NP40 at 23°C for 1 min. Slides were dried in the dark then stained with Vysis DAPI II and the coverslips applied. Images were captured using an Olympus BX60 microscope equipped with a mercury bulb and camera. Images were processed with Cytovision software from Genetix.

### RNA extraction, reverse-transcription and PCR

RNA was isolated from cell pellets using Tri-Reagent (Molecular Research Center, Inc., Cedarlane, Burlington, Canada). Reverse transcription was carried out using 1 μg of RNA, and MMLV (for expression level reactions) or SuperScript II (for sequencing) reverse transcriptase (Life Technologies). Amplification of cDNA by polymerase chain reaction (PCR) was performed using gene-specific primers RBM10F and RBM10RS (exon 4 spanning) [27] (Figure 1Bi) or RBM10v1/v2R (also exon 4 spanning) [20] (Figure 1Bii), NUMBF(exon10): 5′-TAGAAGGGGAGGCAGAGAGC-3′ and NUMBR(exon12): 5′-CTCAGAGGGAGTACGTCTAT-3′ and GAPDH [28] (all primers purchased from AlphaDNA, Montreal, Canada). End-point PCR reaction conditions: (1) 95°C for 5 minutes, (2) gene-specific cycle number (40 cycles for RBM10, 28 and 40 cycles for NUMB, and 19 (Figure 3Bi) or 25 (Figures 1Bii and 3Bii) cycles for GAPDH) of 95°C for 30 seconds, 62°C (RBM10F + RS), 55°C (RBM10F + v1/v2R), 61°C (NUMB), 59°C (GAPDH) for 30 seconds, 72°C for 45 seconds, and (3) 72°C for 10 minutes. The samples were visualized following electrophoresis through a 2% (40 mM Tris-acetate, 10 mM EDTA, pH 8.0) (TAE) agarose gel containing SYBR®safe DNA gel stain (Life Technologies).

For sequencing of full-length RBM10, nested PCR was carried out, using RBM10FNhe1: 5′-CTA GCT AGC TAG TGG CTG GGA AGT GAA ACG GAG CCA GCG-3′ and RBM10RL: 5′-TGG CTG GGG AGT GGG CTG G-3′ primers for Reaction 1 and RBM10FNhe1 and RBM10RHindIII: 5′-CCC AAG CTT GGC TGG GCC TCG TTG AAG CG-3′ primers for Reaction 2, Platinum Pfx Polymerase (Life Technologies) and 5 μl of Reaction 1 as template for Reaction 2. PCR reaction conditions: Reaction 1: (1) 95°C for 2 minutes, (2) 18 cycles of 94°C for 10 seconds, 66°C for 1 minute, 68°C for 3 minutes; Reaction 2: (3) 22 cycles of 94°C for 10 seconds, 62°C for 1 minute, 68°C for 3 minutes, (4) 72°C for 5 minutes.

### Immunoblotting

Western blotting was carried out as previously described [20]. RBM10 antibody was used at a dilution of 1:500. The *in vitro* transcription/translation reactions were carried out using a TNT® T7 Quick Coupled Transcription/Translation Kit (Promega, through Fisher Scientific, Nepean, Canada), and plasmid constructs pcDNA3.RBM10v1 or pcDNA3.RBM10v2 [20].

### Sequencing

Full-length RBM10v1 and RBM10v2 amplicons were separated by agarose gel electrophoresis (note, no full-length RBM10v2 amplicon was detectable in the GLC20 cells) and cDNA was excised using a QIAquick gel extraction kit (Qiagen, Toronto, Canada). DNA quantity (absorbance at 260 nanometers (nm)) and purity (ratio of the absorbance at 260 and 280 nm) were determined using a NanoDrop 2000C spectrophotometer (Fisher Scientific, Ottawa, Canada), and the samples were sent for sequencing. Samples were sequenced, using the Sanger technique, by the MOBIX Lab - DNA Sequencing and Oligo Synthesis Facility (McMaster University, Hamilton, Canada). Internal primers (sequences available upon request) were generated by MOBIX. Bi-directional, overlapping sequence reads of ~600 bp were generated, as detailed in Figure 1D.

### Availability of supporting data

The data set supporting the results of this article is included within the article.

## Abbreviations

RRM: RNA recognition motif
FISH: Fluorescent in situ hybridization
V: Valine
E: Glutamic acid
PCR: Polymerase chain reaction
RT-PCR: Reverse-transcription PCR
nt: Nucleotide
aa: Amino acid
